# Risk factors for oral antimicrobial consumption in Swiss fattening pig farms – a case–control study

**DOI:** 10.1186/s40813-016-0024-3

**Published:** 2016-02-09

**Authors:** Corinne Arnold, Gertraud Schüpbach-Regula, Patricia Hirsiger, Julia Malik, Patricia Scheer, Xaver Sidler, Peter Spring, Judith Peter-Egli, Myriam Harisberger

**Affiliations:** 1 SUISAG, Division SGD, Sempach, Switzerland; 2 Veterinary Public Health Institute, Vetsuisse Faculty, University of Bern, Bern, Switzerland; 3 Department for Farm Animals, Division of Swine Medicine, Vetsuisse Faculty, University of Zurich, Zurich, Switzerland; 4 Berne University of Applied Sciences, HAFL - Agricultural Sciences, Zollikofen, Switzerland

**Keywords:** Pigs, Fattening farms, Risk factors, Antimicrobial consumption, Treatment incidence

## Abstract

**Background:**

Antimicrobial consumption in veterinary medicine is of great importance. Increased awareness by the public and media has led to demands for decreased use of antimicrobials in pigs. This study aimed to identify risk factors for regular oral antimicrobial consumption in Swiss fattening pig farms, and to quantify the amount of antimicrobial active substances administered orally to pigs at the farm level.

**Results:**

A case–control study was performed on 99 fattening farms between May 2014 and January 2015. Seventy-two case farms (with oral group treatment of antimicrobials in at least 50 % of pigs) and 27 control farms (with no regular oral group treatment) were visited once during the study. Data about potential risk factors and antimicrobial consumption were collected by questionnaire. Antimicrobial consumption was recorded and treatment incidence (TI) was calculated for all farms over a one year period. Sulphonamides and tetracyclines were the antimicrobials consumed in the greatest quantity. The median TI for oral antimicrobial use in the case group was 224.7. In the control group, the median TI was 0 for oral antimicrobial use, with values ranging from 0 to 140.1. In a multivariable regression model, seven risk factors associated with regular oral antimicrobial group treatment were identified: mixing pigs from different suppliers within the same pen, absence of a work protocol that ensures treating of healthy pigs before sick pigs, distance to next pig farm < 500 metres, external analysis of production parameters, no availability of dirty visitor boots, the farmer not working on other farms, and no application of homoeopathic agents.

**Conclusions:**

The results of this study point out the importance of increasing farmers’ awareness of good farming practices and biosecurity. Important recommendations for decreasing oral antimicrobial consumption identified by this study include avoiding mixing pigs from different suppliers in the same pen and strictly handling sick pigs after healthy ones. Improvements in these areas could enhance the overall health of pigs and thereby reduce the consumption of antimicrobials on pig farms.

## Background

The amount of antimicrobial agents used in veterinary medicine has been an important topic for many years. The potential risks to public health arising from the high use of antimicrobials in animals have been discussed in various scientific publications as well as in the media [1]. Increased public and media awareness has put increased pressure on farmers and veterinarians to reduce antimicrobial use. In Switzerland, the total quantity of veterinary antimicrobial products sold for use in all animal categories, reached a peak in 2008. Since 2009 the sale of antimicrobials for veterinary use has decreased. In 2013, sales of 53,384 kg of antimicrobials were registered in Switzerland, about two-thirds of which were antimicrobial premixes for administration in feed or water. The total volume sold represents a reduction of 26 % in total Swiss antimicrobial sales compared to 2008 [2]. Although the exact proportion of antimicrobials used in pigs in Switzerland is not known, pigs and cattle were estimated to account for the majority of the veterinary antimicrobial use in 2012 [3]. Swine in Switzerland have a high health status, as the domestic Swiss swine population is free or almost free from several important diseases such as porcine reproductive and respiratory syndrome and enzootic pneumonia [4]. Despite the high health status, antimicrobial use in Switzerland is still relatively high compared to other countries [5]. In swine production antimicrobials are most often applied as group therapy [6] and mostly by oral administration [7, 8]. Previous Swiss studies have reported that the greatest quantities of antimicrobials used during fattening, were used during the first two weeks of the fattening period [9]. Antimicrobials were most frequently administered prophylactically (about 80 % of total amount) [10]. However, prophylactic antimicrobial use has not been shown to decrease mortality, or to reduce the number of therapeutic treatments [10]. Therefore, it could be speculated that there is substantial potential to reduce antimicrobial usage while maintaining a high animal health status.

High antimicrobial consumption is also a concern in other countries. For example, Denmark has introduced a “yellow card”scheme, which imposes restrictions on farmers who exceed predefined levels of consumption [11]. In Germany, a 2014 amendment to legislation introduced a legal requirement for farmers to report antimicrobial use and the antimicrobial usage data is stored in a central database. In addition the responsible local veterinary service has the authority to impose measures on farmers whose antimicrobial usage exceeds defined levels [12]. In the Netherlands an independent institution sets benchmarks for antimicrobial usage, which are re-evaluated on a yearly basis. Farms that exceed these benchmark levels are obliged to decrease their consumption by implementing measures [13]. Since the introduction of this program in 2012, antimicrobial consumption has been reduced by 56 % compared to consumption in 2007. This has been achieved by a combination of compulsory and voluntary actions. The Netherlands has set a new goal of reducing consumption in 2015 by 70 % compared to 2007 [14]. In Switzerland, there is currently no central antimicrobial consumption database that could be used to set benchmarks for antimicrobial consumption.

The identification of risk factors for antimicrobial group treatment in fattening farms is important for developing on farm strategies for reducing antimicrobial use without impairing animal health. However, only a limited number of risk factors for high antimicrobial use have been reported [15–17]. In a study performed in the Netherlands, the risk factors farm system and number of fattening pigs were found to be associated with antimicrobial use on fattening farms [15]. Hybschmann et al. performed a risk factor analysis in Denmark on antimicrobial use for gastrointestinal diseases [16]. Herd size, herd health status and herd type were identified as risk factors [16]. Compared to these countries, herd sizes in Switzerland are smaller and many farms produce for specific pork distribution labels. Farms are often specialised in farrowing or fattening and few produce in a closed system. It is likely that risk factors from studies in other countries will not be valid under these housing and management conditions. The identification of further risk factors would be crucial to support reduction of antimicrobial use. For this reason we conducted a risk factor analysis for fattening pig farms in Switzerland. This study will be relevant to Swiss swine producers and can serve as an example to other countries that have small farms and good general pig health.

The aim of this case–control-study was, to identify risk factors for regular oral antimicrobial treatment in Swiss fattening farms, and to quantify the amount of antimicrobials used at a farm level during a 12-month period.

## Results

### Farm characteristics

A list of 437 potential participants was generated from the Swiss Pig Health Service (SGD) database. Two hundred and sixteen farms were excluded. Of these, 106 farms were excluded before telephone contact, when the list was checked by SGD veterinarians having more current knowledge about these farms, because these farms no longer fulfilled the inclusion criteria. The other 110 farms were excluded after the first telephone interview because they no longer used regular oral antimicrobial group treatments. Of the farms that met the antimicrobial inclusion criteria, 50 % agreed to participate in the study (77 % in the control group, 44 % in the case group). Reasons for not participating included: no interest (total 66 %, control 67 %, case 66 %), farm structure (retirement, ending or already ended pig farming, less than 30 pigs, farmer participates in the study with his other farm) (total 32 %, control 33 %, case 31 %), and other reasons (total 3 %, control 0 %, case 3 %). Eleven of the 110 farms that agreed to participate were excluded retrospectively because they raised pigs for other purposes than fattening.

The final study sample consisted of 99 fattening farms. Ninety-two percent of the farms were members of the SGD. The median weight of pigs at the onset of the fattening period was 25.5 kg (*n* = 97) and the median live weight at slaughter was 109.9 kg (*n* = 87). Herd size varied between 50 and 1300 pig places (median 170). The proportion of total farm revenue represented by swine production varied between 1-100 % (median 25 %). Seventy percent of farmers were between 41 and 60 years old, 14 % were younger than 41, and 16 % older than 60. As a consequence, 68 % of farmers had more than 24 years of experience in pig production. For most farms, the person having the main responsibility for the pigs was the owner of the farm (85 %). Other farms were on a lease arrangement (10 %), partly owned and leased (1 %), or the main responsible person was employed (4 %). Time spent in the pig barn ranged from 0.8 to 28 h per week per 100 pig places with a median of 4.2 h per week per 100 pig places. Daily weight gain of pigs ranged from 675 to 1,014 gram with a median of 808.9 gram (*n* = 80). The median duration of the fattening period was 102 days. Feed conversion (digestible energy pig (DE)/kg) values ranged from 32.5 to 41.0 megajoules DE/kg with a median of 36.0 megajoules DE/kg. However, these data were only available for 70 farms. Mortality rates were less than or equal to 2 % in 69 % of the farms (0–5.5 %, median 1.6 %, *n* = 97).

### Antimicrobial use

A total of 500 kg of active antimicrobial substance was administered orally on the 99 study farms during the 12 months prior to the investigation. Active ingredients used were sulphonamides (305 kg, 61 %), tetracyclines (125 kg, 25 %), trimethoprim (42 kg, 8 %), polymyxin E (8 kg, 2 %), amoxicillin (11 kg, 2 %), macrolides (9 kg, 2 %), and pleuromutilins (0.5 kg, 0.1 %). Five of the twenty-seven control farmers also administered oral antimicrobials, but they all reported treating less than 50 % of their pigs. In both case and control farms, all oral antimicrobials were administered in feed. Results of TI calculations for active substances used orally are presented in Table 1. In the case group, the TI for oral antimicrobials ranged from 29.9 to 418.1 (median 224.7, mean 211.7, SD 101.3). The TI for the control group ranged from 0 to 140.1 (median 0, mean 10.5, SD 29.4). In the case group, 72 % of the farms administered oral antimicrobials to all pigs. The remaining 28 % of case farms orally administered antimicrobials to at least 50 % of pigs during the 12 months prior to the investigation. In the case group, 93 % of the farmers reported that they administered oral antimicrobials mainly for prophylaxis. They usually did not carry out diagnostic examinations prior to treatment, since the treated pigs did not have any abnormal clinical signs. The other 7 % of case farms reported using antimicrobials orally to treat specific conditions (diarrhoea, fever, respiratory symptoms, lameness). Reasons for using oral antimicrobials reported by case group farmers were (multiple answers possible, n in parenthesis): problems occurred during previous fattening period(s) (22), too many different pig suppliers (20), as an insurance policy (16), always used antimicrobials (17), on recommendation (e.g. veterinarian or pig trader) (17) or that an attempt without antimicrobials was not successful (10). All case group farmers were asked if they would also use antimicrobials at the beginning of the production period if they always received pigs from the same single supplier. Forty-four percent answered yes or that they already had only one supplier; forty-eight percent reported that it would probably be possible to work without antimicrobials if they had one supplier, and 8 % were unsure.

**Table 1:** Treatment incidence (TI = Number of animals treated daily with one animal daily dose (ADD) per 1000 pigs) of the active substances for all oral antimicrobials used during the 12 months prior to the investigation of farms. Data are presented for the case group (with oral group treatment of antimicrobials in at least 50 % of pigs) and the control farms (with no regular oral group treatment)

	TI case group (*n* = 80)					TI control group (*n* = 30)				
	Min^1^	Max^2^	Median	Mean	SD^3^	Min	Max	Median	Mean	SD
Sulphonamides	0.0	258.2	101.6	100.6	82.9	0.0	93.4	0.0	4.3	18.1
Tetracyclines	0.0	365.8	0.0	35.5	61.3	0.0	13.5	0.0	0.8	3.0
Trimethoprim	0.0	129.1	34.6	43.9	46.8	0.0	46.7	0.0	1.7	9.0
Polymyxin E	0.0	196.2	0.0	6.9	27.8	0.0	41.1	0.0	2.0	8.2
Amoxicillin (penicillin)	0.0	103.0	0.0	7.8	22.6	0.0	13.3	0.0	0.8	3.0
Macrolides (tylosin)	0.0	117.2	0.0	16.0	31.3	0.0	13.5	0.0	0.8	3.0
Pleuromutilins (valnemulin)	0.0	73.2	0.0	1.0	8.6	0.0	0.0	0.0	0.0	0.0

*Min* ^*1*^ minimum

*Max* ^*2*^ maximum

*SD* ^*3*^ standard deviation

The analysis of antimicrobials administered as injection was performed accordingly. The TI for antimicrobial injections ranged between 0 and 23.3 (median 3.3, mean 5.0, SD 5.5) for the control group and between 0 and 68.5 (median 4.0, mean 5.6, SD 12.5) for the case group.

### Risk factor analysis

The results of screening analyses of risk factors that were associated with the case or control status of the farm (*p*-value <0.1), are presented in Table 2. Factors with a p-value ≥0.1 in the screening included the following topics: health-related data (e.g. estimated disease frequencies or if diagnostic tests had previously been performed), biosecurity (e.g. details about pest control or hand wash facilities), other management practices (e.g. deworming or feeding practices), housing system (e.g. access to outdoor facility or floor types) as well as farm structure and demographic data (e.g. herd size, age of the animal caretaker or if the caretaker owns the farm or is employed).

**Table 2:** All relevant results of the univariable analysis of risk factors (*p* < 0.1) for regular oral antimicrobial group treatment in case farms (more than 50 % of pigs treated) and in control farms (no regular oral group treatment) for Swiss fattening pig farms

Description	Answers	% control group	% case group	p-value (chi^2^ or fisher’s exact)
		*n* = 27	*n* = 72	
Label	Conventional or no label	51.9	37.5	0.0427
	Label 1 (straw, access to outdoor area)	25.9	33.3	
	Label 2 (bedding, access to outdoor area)	14.8	29.2	
	Label 3 = Organic	7.4	0.0	
Renovation of building (pen)	Yes	25.9	58.3	0.0041
	No	74.1	41.7	
Husbandry education	Education 1 (farmer with a certification of achievement)	33.3	33.3	0.0211
	Education 2 (Apprenticeship and further education as pig farm manager)	59.3	38.9	
	Others/no husbandry education	7.4	27.8	
Working on other farms	Yes	25.9	8.3	0.0398
	No	74.1	91.7	
Analysis of production parameters	By farmer (program, computer, written, none)	66.7	30.6	0.0011
	By others (external)	33.3	69.4	
Most frequent cause of death at the onset	Haemorrhagic intestinal syndrome	55.6	33.3	0.0252
	Unknown cause of death	33.3	36.1	
	Other causes	11.1	30.6	
Visitor boots available	Yes, clean	33.3	43.4	0.0084
	Yes, dirty	33.3	15.2	
	No	33.3	41.4	
Production system all-in/all-out	Yes	48.2	77.8	0.0043
	No	51.9	22.2	
Number of suppliers at the same time	One supplier	51.9	19.4	0.0014
	More than one supplier	48.2	80.6	
Pigs originate from same supplier(s)	Yes	59.3	23.6	0.0008
	No	40.7	76.4	
All pigs vaccinated against Lawsonia	Yes	33.3	13.9	0.0287
	No or unknown	66.7	86.1	
Mixing pigs of different suppliers within same pen	Yes	33.3	66.7	0.0028
	No	66.7	33.3	
Work sequence depending on the age	From younger to older pigs	18.5	11.1	0.0115
	No working order (age not considered)	55.6	30.6	
	All pigs having the same age	25.9	58.3	
Cleaning frequency	After each batch: whole barn	44.4	72.2	0.0191
	After each batch: pen(s)	37.0	15.3	
	Less frequent	18.5	12.5	
Heating of barn (before the onset)	Yes	37.0	56.9	0.0775
	No	63.0	43.1	
Work sequence depending on healthy before sick pigs	Yes	59.3	20.8	0.0002
	No	40.7	79.2	
Distance to the next pig farm	<500 metres	25.9	56.9	0.0060
	≥500 metres	74.1	43.1	
Application of homoeopathic agents	Yes	25.9	5.6	0.0084
	No	74.1	94.4	

P-values of the chi^2^ analysis are presented or alternatively for factors with counts ≤5 for a group, results of the fisher’s exact testing are shown

Variables that were included in the final multivariable logistic regression model are presented in Table 3. Seven factors associated with regular use of antimicrobials in feed were identified. A farmer mixing pigs from different suppliers within the same pen had a 4 times higher odds of being in the group with regular oral antimicrobial use than farmers that did not mix pigs. Proximity to other pig farms was also found to be a potential risk. The odds of being in the case group were almost 10 times greater among farms having a neighbouring farm within a 500 meter radius. Farmers, who did not follow a specified work sequence that included managing healthy pigs before sick pigs, had approximately 16 times higher odds of being in the case group than farmers who followed such a sequence. Farmers who did not use homoeopathic agents had about 10 times higher odds of being in the group with regular antimicrobial use than farmers who used homoeopathic agents. Working on other farms had a protective effect, as farmers who worked on other farms were less likely to be in the case group (odds ratio = 0.05). The analysis of performance data by the farmer (program, computer, written, or none) was also found to be protective (odds ratio = 0.12). The presence of dirty visitor boots on farms was protective (odds ratio = 0.06) when compared to the absence of visitor boots.

**Table 3:** Results of the multivariable logistic regression model for the risk factor analysis for oral antimicrobial use in Swiss fattening pig farms

Description	Answers	*p*-value model	OR^b^	95 % CI^c^
Work sequence depending on healthy before sick pigs (Ref.^a^ Yes)	No	<0.01	16.68	3.4-81.8
Working on other farms (Ref. No)	Yes	<0.01	0.05	0.006-0.4
Distance to the next pig farm (Ref. ≥ 500 metres)	<500 metres	0.01	9.88	1.7-57.1
Visitor boots available (Ref. No boots available)	Yes, clean	0.97	1.03	0.2-4.7
	Yes, dirty	0.01	0.06	0.006-0.5
Analysis of production parameters (Ref. by others (external))	By farmer (program, computer, written, none)	0.01	0.12	0.02-0.6
Application of homoeopathic agents (Ref. Yes)	No	0.02	10.49	1.4-78.8
Mixing pigs of different suppliers within the same pen (Ref. No mixing)	Yes	0.05	4.16	1.0-17.4

^a^Ref.: reference group

^b^OR: odds ratio

^c^CI: confidence interval

## Discussion

In this study risk factors for regular oral antimicrobial use on Swiss fattening pig farms were identified and the amount of antimicrobials used at a farm level was quantified. Participation rates were 77 % for the control, and 44 % for the case groups (in total 50 %). The difference in participation between the two groups may have been influenced by the study design. All of the case farms were also enrolled in an additional longitudinal study for the FitPig project, which required at least one additional farm visit. Therefore, more case than control farmers elected not to participate, because the farmers considered the extra visit(s) to be too time-consuming. For this reason voluntary participation by farmers may have introduced a bias into this study. Having a biased sample may have resulted in an underestimation of antimicrobial consumption. This bias could be stronger in the case farms, because the participation rate was lower in this group.

### Antimicrobial use

Sulphonamides and tetracyclines accounted for the major proportion of orally administered substances. This is in agreement with findings reported in an earlier study performed in Switzerland [18]. The usage patterns reported in this study differ from those found in other countries, where tetracyclines are more commonly used than sulphonamides [13, 19, 20]. A possible explanation could be that Swiss pig farmers were discouraged from using certain antimicrobials, including tetracyclines, in the past and SGD members are still discouraged from using certain antimicrobials, including tetracyclines, without carrying out further diagnostics. This is done to prevent antimicrobial administration from masking of clinical signs of economically important diseases such as enzootic pneumonia or swine dysentery which are systematically monitored by the authorities or the SGD health programme (personal communication Y. Masserey, head of regional SGD office).

To allow a standardized comparison of usage among farms, the TI was calculated. The TI has been used in previous studies to describe antimicrobial use [7, 21, 22]. In this study there was a wide range of farm level TI values. This was likely due to the definition of case and control farms used in this study. For this reason, the TI’s may not be representative of Swiss fattening farms and generalizing to the population of Swiss fattening farms should be done with caution. On the farms in this study, there was no evidence that the control group had to compensate for the lower use of oral antimicrobials using a larger quantity of parenteral use of antimicrobials.

A comparison to the TI’s found in other studies was not carried out, because methods for calculating TI’s has not been standardized across countries and the recommended animal daily dose (ADD) varies between countries. An international working group is currently developing a list of ADD’s that is valid for international use [23]. However, at this time the list does not include all antimicrobial products used on farms in this study. Other differences between countries include variation in methods for estimating days at risk and for kg of pig treated [7, 21]. In a recent study Moreno 2014 [24] reported that different values were used in almost all studies. Setting standard values is crucial for enabling comparisons between countries.

Five of the twenty-seven control farms used oral antimicrobials. Even though the TI’s on these farms were low, there was a small number of control farms that had higher TI values than some of the case farms. On control farms oral administration mostly took place later in the fattening period and for therapeutic treatment of sick animals. These animals had a higher average weight and therefore more active substance was required to treat them. This could not be accounted for in the calculation of the TI used in this study, because the same standard pig weight was used for all farms. Finally, differences in TI may have been due to farmers administering antimicrobials at lower than recommended dosages, or for shorter treatment periods than recommended. There were differences in data quality between farms because some farmers did not report the number of animals treated or the administered dosage. For these reasons it was possible to calculate TI based only on amount of active substance used, rather than calculating daily doses used.

### Risk factor analysis

In this study, seven risk factors for increased oral antimicrobial use at the beginning of the fattening period were identified in Swiss fattening farms. A higher risk for oral antimicrobial use was found on farms mixing pigs from different supplying farms within the same pen. It is very likely that pigs from different farms were exposed to different pathogens. Transport in combination with formation of new groups [25] can cause stress for the animals, and can increase the risk of disease occurrence. This first risk factor was associated with the number of supplying farms, and whether the suppliers changed over time. These were also identified as risk factors in other studies [10, 26]. However, these were not in the final multivariable model in this study. A work sequence, in which sick animals were handled before healthy ones, was also a risk factor for regular antimicrobial use. This risk factor could be related to differences in the awareness of the importance of good management practices between case and control farms. The difference in awareness might also explain why other risk factors such as not working on other farms, external analysis of production parameters and no availability of dirty visitor boots were associated with increased oral antimicrobial use. Working on other farms may support the exchange of knowledge and increase the general awareness for good biosecurity. The factor of dirty visitor boots being of a lower risk than no visitor boots cannot be easily explained. It is possible that in a retrospective study design as applied in the present investigation, some factors in statistical analysis could have been found by coincidence. The higher risk for farms located in a radius of less than 500 metres to other pig farms could be due there being a higher probability of pathogen transfer over short distances. This factor was already described by van der Fels-Klerx et al. [15] as potential risk factor. Farms administering homoeopathic agents had a lower risk for regular antimicrobial consumption. It may be possible that farmers in the control group were looking for alternative substances to antimicrobials and used homoeopathic agents instead. An indirect association may be more likely than direct causality for some risk factors. For example external analysis of performance data could be interpreted as indicator for the professional attitude of the farmers. Some of the factors not being part of the multivariable model in this study have been reported as risk factors in other studies. The size of the farm [15, 16] had no significant effect in this study. In another study performed in Switzerland, the sanitary break (time period when no pigs are in the barn or pen, before the next group of pig arrives), and not consequently practicing all-in-all-out were identified as risk factors, but were not part of the multivariable model of this study [10].

## Conclusion

In this study, risk factors for increased oral antimicrobial use on Swiss fattening pig farms were identified. An important recommendation to decrease oral antimicrobial consumption would be to avoid mixing pigs from different suppliers in the same pen. Additionally, more attention should be paid to the work sequence. Sick pigs should be handled after handling healthy ones. This study suggests that it would be important to increase the awareness of the farmers of the value of good farming practices, biosecurity and herd health. Improving the overall health of the pigs would help to reduce the consumption of oral antimicrobials on fattening pig farms.

## Methods

### Data collection

#### Study design

A case–control study was performed with 99 fattening pig farms in Switzerland. Each farm was visited once. The control group consisted of 27 and the case group of 72 participating farms. The difference in group sizes was due to a follow-up intervention study in which only the farms of the case group participate. The follow-up study is a controlled field trial with the aim to reduce antimicrobial usage in farms with routine use of oral antimicrobials. The sample size for our study was calculated to detect an odds ratio of 3.5 with a power of 80 % and a significance level of 5 %, using the software PASS 12 (Hintze, J. (2013). PASS 12. NCSS, LLC. Kaysville, Utah, USA. www.ncss.com). The following inclusion criteria were used to define and select case farms: oral antimicrobial group treatment taking place at the beginning of the fattening period had to be administered to at least 50 % of all pigs during the previous twelve months. The control group included farms without routine oral group treatment or, farms where oral antimicrobials were administered to less than 50 % of all pigs in the last 12 months. The minimum farm size was 30 pig places. The person mainly responsible for the pigs had to have adequate German language skills to be able to answer the questionnaire accurately.

#### Recruitment

A list of potential case and control farms was generated from the database of the SGD, where information on oral antimicrobial treatments was available from January 2011 to August 2014. About 60 % of the Swiss fattening farms are members of the SGD (personal communication HP. Keller, CEO SUISAG). Farmers joining the SGD benefit from a health programme with the main goal of preventing the spread of economically important diseases. The programme mainly consists of certifying farms according to their health status and rules for pig trading. Regular farm visits by a veterinarian are also a key element of the programme. All farms fulfilling the study inclusion criteria were extracted from the SGD database. To achieve a broader representation of the Swiss population of pig fattening farms, an additional effort was made to recruit farmers who were not members of the SGD. Veterinarians of the Swiss Association of Pig Medicine were asked to identify farms fulfilling the inclusion criteria. From the list of both groups of farmers, case and control farms were randomly selected until a sufficient sample size of farms, fulfilling all inclusion criteria, was achieved. Initially, a letter was sent to all farmers, informing them about the upcoming project. Farmers were subsequently contacted by telephone, inclusion criteria were verified, and they were asked to participate. All participants were recruited and visited between May 2014 and January 2015. All visits were performed by 3 veterinarians working on the project.

#### Materials

Data were collected using two questionnaires. One was sent to the farmers before the farm visit and the second was completed during the farm visit. Questionnaires were designed by a group of experts (pig veterinarian, epidemiologists, and agronomist) to align with the results of former studies [10, 22]. Questionnaires included the following topics: farm structure and details about the farmer, performance data, housing, management, food and water supply, health of pigs, biosecurity and antimicrobial use. Draft questionnaires were evaluated by a social scientist with experience in questionnaire design and the questionnaire was pretested on 2 farms. The results of the pre-test were not included into the final analysis. Questionnaires are available on request from the authors (in German). Data on the antimicrobial consumption within the last twelve months were extracted either from the farm inventory or the treatment journal, prescription forms or, if these were not available, from the veterinarian’s invoices.

### Data analysis

Statistical analysis was performed using NCSS 9, NCSS, LLC. Kaysville, Utah, USA. Descriptive statistic was carried out for potential risk factors and antimicrobial usage. For antimicrobial consumption, the amount of active substance used was calculated for each substance separately as the product of number of units (e.g. ml, mg) administered and the weight of active ingredient per unit (e.g. mg/ml or mg/kg). To enable a comparison of the antimicrobial use between farms, the TI was chosen as a measure of usage [7, 21, 22]. The TI was calculated by dividing the amount of active substance used (mg) through the product of: the ADD, the days at risk and kg weight of pigs. The outcome was then multiplied by 1000. The TI is a measure of the number of animals treated daily with one ADD per 1000 pigs [21]. Information about the ADD for each product was extracted from the Swiss on-line database of pharmaceutical products [27]. For products with a range of recommended doses, the lowest value was used for orally administered antimicrobials. For antimicrobials administered by injection, the highest value was used. This protocol was based on previous studies demonstrating that oral antimicrobials are often administered at below the recommended dosage, and injections are more likely to be administered at above the recommended dosage [7, 21]. To estimate the number of days at risk, the median length of the fattening period was taken from the data collected by questionnaire. The kg weight of pigs was calculated as the total number of pigs produced in one year multiplied with the average weight at the beginning of fattening. Since weight was not available on all of the study farms, the average weight at the beginning of fattening (26.8 kg) in 2014 was obtained from one of the main Swiss pig marketers (personal communication M. Reich, Anicom, Switzerland).

All data were entered into NCSS 9 and checked for plausibility and counts per group. Continuous data, where a non-linear relationship with the outcome was expected, were grouped into several categories. Univariable screening of all potential risk factors was performed using chi-squared tests. For factors that contained less than 5 counts per group, fisher’s exact tests were performed. Variables with p-values < 0.1 were considered for entry into the multivariable logistic regression model. All potential risk factors were screened for correlation among each other. If a high correlation between two variables was observed (phi > 0.7), the biologically more meaningful variable was selected for the model. The multivariable logistic regression model was built with a stepwise forward selection procedure. Only variables significantly associated with being a case farm (p < 0.05) were retained in the model. Biologically meaningful interactions between the risk factors were tested, but none of them was significant.
